# Distribution Characteristics of Low-Molecular-Weight Organic Acids in Reclaimed Soil Filled with Fly Ash: A Study

**DOI:** 10.3390/toxics12050312

**Published:** 2024-04-26

**Authors:** Yonghong Zheng, Yue Wu, Zhiguo Zhang, Fangling Chen, Qingbin Ma, Zihao Kong, Ying Ma

**Affiliations:** 1 School of Earth and Environment, Anhui University of Science and Technology, Huainan 232001, China; zyhaust@aust.edu.cn (Y.Z.); 13966466580@163.com (Y.W.); 17355481835@163.com (F.C.); 13721179192@163.com (Q.M.); kzh1195948632@163.com (Z.K.); yingma0514@163.com (Y.M.); 2 Academician Workstation in Anhui Province, Anhui University of Science and Technology, Huainan 232001, China

**Keywords:** available nutrient contents, reclamation soil, soil nutrients, LMWOAs, high performance liquid chromatography

## Abstract

This study aims to assess the contents of different kinds of low-molecular-weight organic acids (LMWOAs) in reclaimed soil filled with fly ash in the Huainan mining area in China using high-performance liquid chromatography (HPLC). Using a mobile phase consisting of 0.1% phosphoric acid and acetonitrile in a volume ratio of 98:2, the detection was performed at a wavelength of 210 nm for 15 min. In addition, a cluster analysis was performed on the detected LMWOAs in the reclaimed soil. The correlations between the LMWOA and nutrient contents in the reclaimed soil were also analyzed. In total, eight and seven LMWOAs were detected in the reclaimed soil and filled fly ash, respectively. In contrast, no LMWOAs were detected in the fresh fly ash from a thermal power plant. The order of total LMWOA contents at different sampling points followed the order of farmland control soil > 1# (*Triticum aestivum*) > 4# (*Phragmites australis*) > 5# (*Vigna radiata*) > 2# (*Sorghum bicolor*) > 3# (*Tamarix ramosissima*) > fly ash-filled soil. The farmland control soil and fly ash-filled soil exhibited the highest and lowest LMWOA contents of 648.22 and 85.09 μg·g^−1^, respectively. The LMWOA contents in the reclaimed soil followed the order of oxalic acid > tartaric acid > malonic acid > lactic acid > acetic acid > citric acid > propionic acid > succinic acid. Indeed, oxalic acids exhibited the highest total amount of 1445.79 μg·g^−1^ and succinic acids exhibited the lowest total amount of 6.50 μg·g^−1^. The LMWOA contents in the reclaimed soil decreased with increasing soil depth, showing statistically significant differences between the 0–10 and 10–40 cm soil layers (*p* < 0.05). According to the obtained clustering results, the detected LMWOAs can be divided into two categories. The first category consisted of oxalic acid, while the second category included the remaining LMWOAs. The soil LMWOA contents of 4# (*Phragmites australis*) and 5# (*Vigna radiata*) were significantly different from those at the other sampling points. According to the Pearson correlation analysis results, the occurrence and characteristics of the soil LMWOAs can be controlled by regulating the pH values and available nutrient contents in the soil, thereby improving the eco-environmental conditions of the reclaimed rhizosphere.

## 1. Introduction

Soils provide essential nutrients for plants, thereby affecting plant growth and development. Organic acids in soils are one of the important factors restricting soil nutrient availability and affecting plant growth [1,2]. Organic acids are functional organic compounds widely present in soil and litter layers [3]. The classification includes high-molecular-weight organic acids and low-molecular-weight organic acids (LMWOAs), with the former containing more than three carboxyl groups, encompassing humic acid and fulvic acid [4] and the LMWOAs weighing less than 250 Da. They typically contain one or more hydroxyl, carboxyl, and amino groups, which can stimulate plant growth and serve as growth regulators. LMWOAs in soils are mainly derived from plant root exudates, animal and plant decompositions, and microbial metabolisms. In soils, the main types of LMWOAs include more than 10 types, including oxalic acid, citric acid, and malic acid [4,5,6]. These LMWOAs can affect soil chemical and biological processes through complex mechanisms, such as chelation and ligand exchange. They can also influence soil physicochemical properties, promote plant uptake of soil nutrients, and reduce the toxicity of heavy metals and other toxic substances in plants [6,7,8,9]. Due to the unique molecular structure and charge characteristics of LMWOAs, they play a crucial role in improving soil nutrient availability release [10,11,12], soil heavy metal activation [13,14,15], and microbial community impacts [16]. In addition, the topic has attracted considerable attention in interdisciplinary research, including soil science, plant nutrition, and ecological research both in China and around the world.

Fly ash is one of the main waste materials derived from coal combustion in thermal power plants. It is a mixture of coal ash and slag, with granular particle forms ranging from 0.1 to 5.0 mm, and its accumulation can increase sharply [17]. Fly ashes are characterized by large specific surface areas and strong water retention abilities. Furthermore, they may contain some trace elements essential for plant growth and development, such as iron (Fe), zinc (Zn), copper (Cu), aluminum (Al), and boron (B) [18,19]. Therefore, using fly ash as filling materials in collapsed areas of coal mines can not only restore the ecological functions of collapsed lands but also reduce environmental pollution caused by fly ash generation. However, fly ash-based land restoration may result in low soil fertility levels and high soil alkalinity due to the relatively low nutrient contents in fly ashes [20,21]. The characteristics of LMWOAs and their relationships with soil nutrients in fly ash-filled coal mine reclamation areas in China and worldwide, as well as their impact mechanisms, are still unclear. In this context, the present study aims to: (1) determine the types and contents of LMWOA acids in reclaimed soil, (2) evaluate the correlations between LMWOAs and soil nutrient indicators in the reclaimed soil, and (3) provide a theoretical basis for improving soil fertility and controlling pollutants in fly ash-filled soil.

## 2. Materials and Methods

### 2.1. Overview of Study Area

The Huainan mining area is located in the central-northern part of Anhui Province, within the longitude and latitude ranges of 116°21′21″ E–117°11′59″ E and 32°32′45″ N–33°00′24″ N, respectively. It extends 180 km from east to west and 15–25 km from north to south, covering a total area of approximately 3200 km^2^. The region is characterized by a monsoon-warm temperate semi-humid climate, with dominant southeast winds and an average annual temperature of 15 °C. The average annual precipitation of the Huainan is 1011.7 mm. The ash storage yard of a power plant in the Huainan mining area covers an area of about 15,000 m^2^. The land of the ash storage yard was reclaimed in October 2018. The final surface soil thickness after leveling was about 40–50 cm to promote natural vegetation growth after the reclamation. This reclamation not only restored arable land but also addressed a series of environmental problems caused by the open-air fly ash storage of the power plant. The predominant chemical composition of fly ash in this mining area consists primarily of oxides, with SiO_2_ accounting for 54.63%, Al_2_O_3_ accounting for 28.14%, CaO accounting for 1.25%, and Fe_2_O_3_ accounting for 3.76%.

### 2.2. Sample Collection and Pretreatments

In this study, five sampling points and one farmland control point were selected in October 2021 based on the dominant plants in the study area. The corresponding plants of each sampling point are shown in Table 1. The control plots were ordinary agricultural land where wheat had been grown. The soil collected comes from the rhizosphere, each sampling point consisted of three 1 m × 1 m sampling plots. Soil samples were collected layer-by-layer at a depth of 10 cm using soil augers, mining to the end of the fly ash layer. In addition, fly ash samples were collected from the bottom layer. The collected soil samples from the three plots were mixed, and then the required amount was taken using the quartering method and placed in a numbered polytetrafluoroethylene sample bag. A global positioning system (GPS) instrument was employed to locate the sampling points. A portion of fresh fly ash from the power plant in the mining area was obtained.

The collected samples are divided into two parts. One part was used to determine the LMWOA contents. Specifically, the soil samples were rapidly stored in an insulated box containing ice bags at a temperature of 4 °C. They were subsequently sent to the laboratory and stored in a refrigerator at −20 °C. The second part of the soil samples were air-dried on a sample air-drying tray before removing dried plant roots and other debris from the samples. The air-dried samples were ground in an agate mortar, sieved through nylon meshes of different sizes, sealed, and stored in labeled and numbered self-sealing bags. These samples were analyzed for soil physicochemical properties.

### 2.3. Analytical Instruments and Reagents

Japanese Hitachi L-2000 high-performance liquid chromatography (HPLC), Sepax Bio-C18 analytical column (4.6 mm × 250 mm, 5 μm), Japanese Shimadzu AUW220 electronic balance, Beckman Allegra-64R high-speed freezing centrifuge, and SHZ-82A constant-temperature water bath incubator (Tianjin Tianjing Experimental Instrument Factory, Tianjin, China) were employed in this study.

Nine chromatographically pure standard organic acids, purchased from Tianjin Guangfu Reagent Research Institute, were used in this study, namely oxalic acid, tartaric acid, formic acid, malonic acid, lactic acid, acetic acid, citric acid, succinic acid, and propionic acid. In addition, other chromatographically pure acids were also employed, which are acetonitrile (TEDIA, Cincinnati, OH, USA) and phosphoric acid (Tianjin Damao Chemical Reagent Factory, Tianjin, China). Ultrapure water was used throughout the analyses.

### 2.4. Organic Acid Determination Method and Liquid Chromatographic Conditions

In this study, 5 g of the fresh soil sample was accurately weighed and placed in a 50 mL centrifuge tube and then 10 mL of 0.1% H_3_PO_4_ aqueous solution was added. The mixture was stirred with a glass rod to evenly mix the soil solution, oscillated on a reciprocal shaker for 10 min, and centrifuged at 3000 rpm for 5 min. The solution was subsequently filtered through a 0.45 μM microporous filter membrane. The compositions and contents of the LMWOAs were determined using an L-2000 high-performance liquid chromatography. The types of the selected LMWOAs were determined by comparing their retention times with those of known standards. Each analysis was repeated three times.

**Figure 1:**
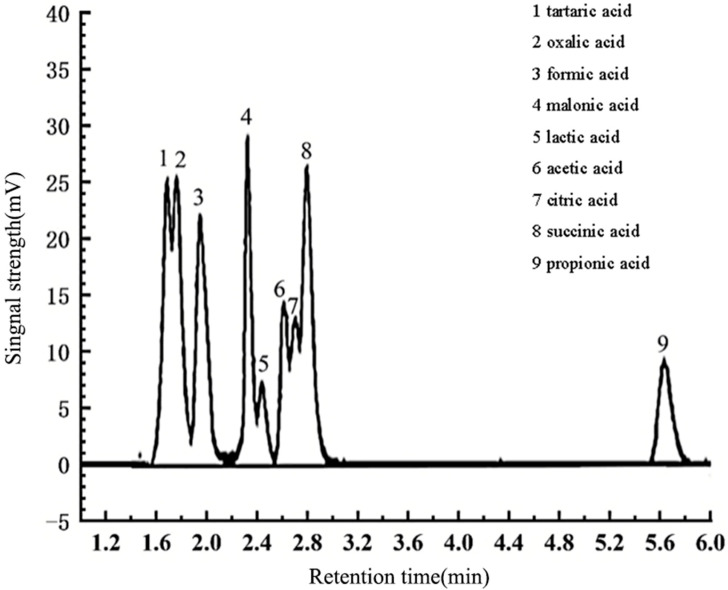
Chromatograms of mixed standard solution of nine organic acids.

The mobile phase consisted of 0.1% phosphoric acid and acetonitrile (98:2 *v*/*v*), with a 0.45 μM microporous filter membrane filter. In addition, ultrasonic degassing was applied prior to the chromatography analysis. The flow rate was 1 mL·min^−1^, with column temperature, detector wavelength, injection volume, and detection time of 35 °C, 210 nm, 20 μL, and 15 min, respectively. Qualitative analysis was conducted based on the chromatographic retention time of the standard substance, and the enhanced integration method of Empower chromatography workstation was used to determine the peak area for quantification. The chromatograms of the nine LMWOA standard solutions are shown in Figure 1.

### 2.5. Statistical Analysis 

The collected data were statistically analyzed using SPSS 25.0 and Excel 2016 (version number:1707) software. The statistical significance of the differences between treatments was assessed using the single sample *t*-test. In addition, the relationships between the changes in organic acid content were analyzed using Pearson correlation analysis. Origin 2022 statistical software (version number: 9.9.0.225) was employed to draw line charts, cluster heatmaps, and Pearson correlation graphs. 

## 3. Results and Analysis

### 3.1. Contents and Species of Low-Molecular-Weight Organic Acids in Reclaimed Soil

According to Table 2, a total of eight LMWOAs were detected in the reclaimed soil, indicating the lack of formic acid. On the other hand, the LMWOAs were not detected in the fresh fly ash of the power plant. According to the observed total LMWOA content at the seven sampling points, the highest total LMWOA content in the farmland control soil was 648.22 μg·g^−1^, while the lowest total LMWOA content in the fly ash-filled soil was 85.09 μg·g^−1^. The total LMWOA content followed the order of farmland control soil > 1# (*Triticum aestivum*) > 4# (*Phragmites australis*) > 5# (*Vigna radiata*) > 2# (*Sorghum bicolor*) > 3# (*Tamarix ramosissima*) > fly ash-filled soil. Oxalic acid and succinic acid exhibited the highest and lowest total contents at the seven sampling points of 1445.79 and 6.50 μg·g^−1^, respectively. The contents of the LMWOA types followed the order of oxalic acid > tartaric acid > malonic acid > lactic acid > acetic acid > citric acid > propionic acid > succinic acid.

**Figure 2:**
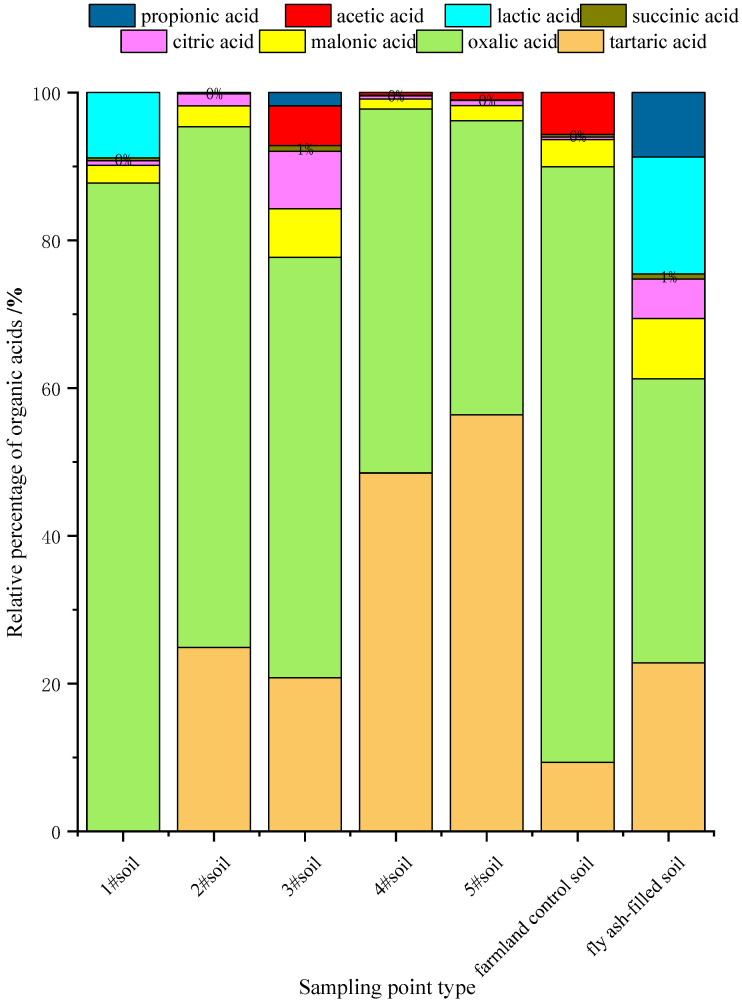
Types and content of organic acid in soil.

The results revealed significant differences in the compositions, and contents of the eight LMWOAs at the seven sampling points were observed (Figure 2). Among them, oxalic acid and tartaric acid were the dominant organic acid types in the samples. The oxalic acid contents followed the order of farmland control soil (80.58%) > 2# *Sorghum bicolor* (70.48%) > 1# *Triticum aestivum* (64.09%) > 3# *Tamarix ramosissima* (56.89%) > 4# *Phragmites australis* (49.24%) > 5# *Vigna radiata* (39.80%) > fly ash-filled soil (38.41%). The tartaric acid contents followed the order of 5# *Vigna radiata* (56.39%) > 4# *Phragmites australis* (48.52%) > 1# *Triticum aestivum* (26.98%) > 2# *Sorghum bicolor* (24.88%) > fly ash-filled soil (22.83%) > 3# *Tamarix ramosissima* (20.81%) > farmland control soil (9.38%). In this study, up to seven LMWOAs were detected in six soil samples from sites with 3# *Tamarix ramosissima*, while at least five LMWOAs were detected at sites with 2# *Sorghum bicolor*. Among them, the malonic, citric, succinic, lactic, acetic, and propionic acid contents were relatively low, not exceeding 10% of the total organic acid amount. On the other hand, seven LMWOAs were detected in the fly ash-filled soil, with the exception of acetic acid. Except for oxalic acid, tartaric acid, and lactic acid, the malonic, citric, succinic, and propionic acid contents did not exceed 10% of the total organic acid content.

### 3.2. Characteristics of Low-Molecular-Weight Organic Acid Contents in Reclaimed Soil

**Figure 3:**
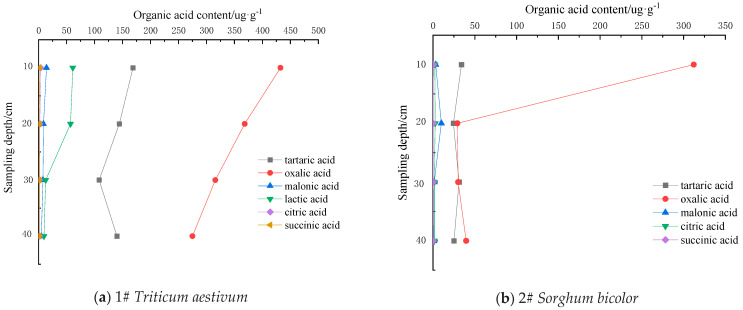

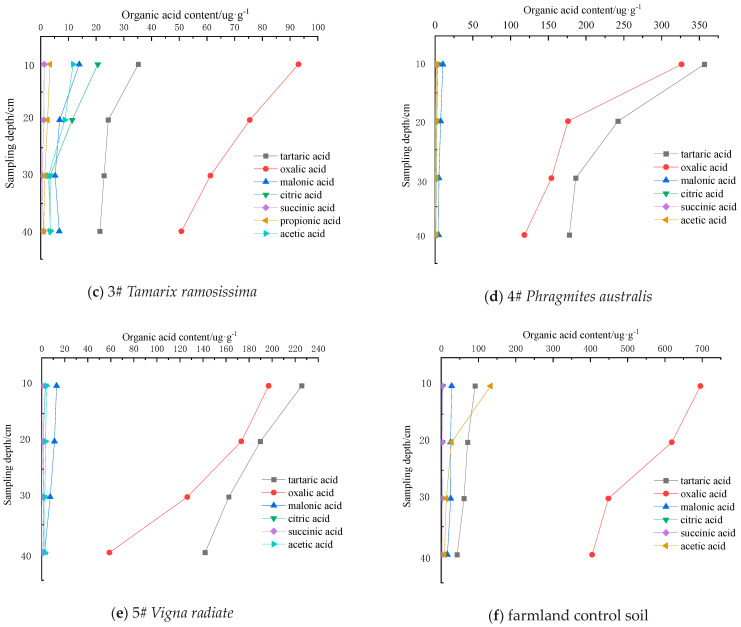
Distribution characteristics of organic acids in soil profiles.

According to Figure 3, the LMWOA contents in the reclaimed soil layers decreased with increasing soil depth. Indeed, the results revealed statistically significant differences in the LMWOA contents between the 0–10 and 10–20, 20–30, and 30–40 cm soil layers. The LMWOA content in the soil profile of *Triticum aestivum* at sampling point 1 followed the order of oxalic acid > tartaric acid > lactic acid > malonic acid > citric acid > succinic acid (Figure 3a). The citric and succinic acid contents did not exhibit significant changes with soil depth. The LMWOA contents in the soil profile of Sorghum bicolor at sampling point 2 showed the order of oxalic acid > tartaric acid > malonic acid > citric acid > succinic acid (Figure 3b). Among them, the oxalic acid content in the 0–10 cm soil layer was 312.21 μg·g^−1^, which was 10.69 times higher than that observed in the 10–20 cm soil layer. On the other hand, there was a lack of significant differences in the contents of the remaining LMWOAs between the soil layers. Up to seven LMWOAs were detected in the soil profile of *Tamarix ramosissima* at sampling point 3 (Figure 3c). The order of the LMWOA contents in the 0–10 cm soil layer was oxalic acid > tartaric acid > citric acid > malonic acid > acetic acid > propionic acid > succinic acid. The single sample T-test results showed a lack of significant differences in the LMWOA content between the 10–20, 20–30, and 30–40 cm soil layers (*p* > 0.05), indicating that the LMWOA content did not change significantly with soil depth.

The LMWOA content in the soil of *Phragmites australis* at sampling point 4 followed the order of tartaric acid > oxalic acid > malonic acid > citric acid > acetic acid > succinic acid (Figure 3d). Indeed, the dominant LMWOAs (tartaric and oxalic acids) showed decreasing trends with increasing depth. On the other hand, the citric, acetic, and succinic acid contents were relatively low without exhibiting significant differences between the soil layers (*p* > 0.05), indicating a lack of significant changes in their contents with soil depth. The LMWOA contents in the soil of *Vigna radiate* at sampling point 5# followed the order of tartaric acid > oxalic acid > malonic acid > acetic acid > citric acid > succinic acid (Figure 3e), of which tartaric acid and oxalic acid showed decreasing trends of their contents with increasing soil depth. The single sample T-test results showed a lack of significant differences in the malonic, acetic, citric, and succinic acid contents between the soil layers (*p* > 0.05), indicating that the contents of these LMWOAs did not change significantly with increasing depth. The LMWOA contents in the 0–10 cm layer of the farmland control soil at sampling point 6 followed the order of oxalic acid > tartaric acid > acetic acid > malonic acid > citric acid > succinic acid (Figure 3f). In addition, the dominant oxalic acid showed significant decreasing trends with increasing soil depth. On the other hand, the single sample T-test results showed a lack of significant differences in the contents of organic acids except oxalic acid and acetic acid in soil (*p* > 0.05), indicating that the contents of the acids did not change significantly with increasing soil depth. 

### 3.3. Cluster Analysis of Low-Molecular-Weight Organic Acids in Reclaimed Soil

**Figure 4:**
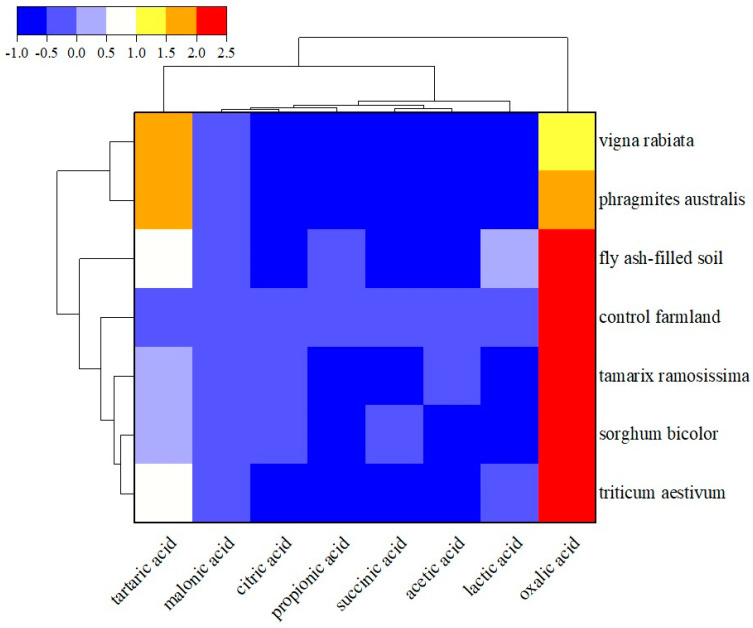
Clustered heat map of organic acids in soil.

Heat maps are two−dimensional visualization graphs presenting comprehensively and intuitively multidimensional data. In these graphs, color gradients represent the magnitude of values. On the other hand, hierarchical cluster analysis is based on the similarity between variables or samples. Specifically, samples with similar characteristics are grouped in the form of clusters, facilitating subsequent correlation analyses between various indicators [22]. In this study, the LMWOA content in the soil samples was analyzed using multivariate statistics. A clustering heat map (Figure 4) was generated using the Z-score normalization method in Origin 2022 software (version number: 9.9.0.225).

According to the vertical clustering tree in the heat map, the LMWOAs can be classified into two major categories. The first category included oxalic acid, with a significantly higher content than the central value, demonstrating the dominance of this LMWOA in the soil. The second category included the remaining detected LMWOAs, namely tartaric, malonic, citric, succinic, propionic, acetic, and lactic acids. These seven organic acids (excluding tartaric acid) exhibited significantly lower contents than oxalic acid in the soil, except lactic acid, which showed a higher content in the fly ash−filled soil than the central value.

The horizontal clustering tree of the heat map showed that the sampling soil points can be classified into two major categories. The first category included sampling points 4# (*Phragmites australis*) and 5# (*Vigna radiata*). The soils of these sampling points were characterized by the presence of tartaric acid and oxalic acid, as well as other LMWOAs with contents lower than the central value. The second category included sampling points 1# (*Triticum aestivum*), 2# (*Sorghum bicolor*), and 3# (*Tamarix ramosissima*), as well as the farmland control soil and the fly ash−filled soil. These sampling sites exhibited high oxalic and tartaric acid contents, of which oxalic acid showed the highest content. Whereas the remaining six LMWOAs showed contents below the central value.

### 3.4. Correlations between Soil Nutrients and Organic Acids in Reclaimed Soil

In this study, the soil pH value of the reclamation area ranged from 7.27 to 7.82 (Table 3). In fact, the pH values indicated a transition from neutral to weak alkaline soils according to the soil pH grading standard [23]. In addition, the results revealed an increasing trend of the soil pH value with increasing soil depth, which is consistent with the results found by Hu et al. [24]. The pH value of the farmland control soil ranged from 6.57 to 7.41, indicating relatively neutral soil. In addition, the pH value of the farmland control soil exhibited an increasing trend with increasing soil depth. The organic matter contents in the reclaimed soil were less than 30 g·kg^−1^, suggesting a relatively low−moderate soil fertility level according to the soil nutrient grading standards of the section national survey of China. Nevertheless, the organic matter contents in the reclaimed soil were slightly higher than those observed in the farmland soil. The organic matter contents showed a decreasing trend with increasing soil depth. Compared to farmland soil, the available potassium contents in the soil of the reclamation area were slightly lower, measuring less than 150 mg·kg^−1^, indicating a low–moderate soil fertility level. Moreover, the available potassium content showed a decreasing trend with increasing soil depth. The available phosphorus content in the soil of the reclamation area were low (less than 10 mg·kg^−1^), indicating a relatively poor soil fertility level. In addition, the results showed a decrease in the available phosphorus content with increasing soil depth. The alkali−hydrolyzable nitrogen contents in the soil of the reclamation area were less than 90 mg·kg^−1^, suggesting a relatively poor fertility level. The available phosphorus and alkali−hydrolyzable nitrogen contents in the reclaimed soil were relatively lower than those observed in the farmland soil. These findings are consistent with the vertical distributions of the soil organic carbon and nutrient contents in topsoil revealed by Yan et al. [25]. Overall, there were relatively low organic matter, available phosphorus, available potassium, and alkali−hydrolyzable nitrogen contents in the reclaimed soil. On the other hand, the available potassium and phosphorus contents in the fly ash were comparatively higher, thereby exhibiting positive fertilization effects on the reclaimed soil to some extent.

In this study, Pearson correlation analysis was performed to assess the relationships between the reclaimed soil nutrient indicators and soil LMWOAs. Table 4 indicated that the reclaimed soil pH values were negatively correlated with the oxalic, malonic, citric, and acetic acid contents (*p* < 0.01). The available potassium contents exhibited a significant positive correlation with citric acid (*p* < 0.01), as well as significant negative correlations with oxalic acid (*p* < 0.01) and tartaric acid (*p* < 0.05). The organic matter contents showed significant positive correlations with lactic acid (*p* < 0.01), malonic acid (*p* < 0.05), and citric acid (*p* < 0.05). The alkali−hydrolyzable nitrogen contents were positively correlated with malonic acid (*p* < 0.01), negatively correlated with tartaric acid (*p* <0.01), and positively correlated with oxalic acid (*p* < 0.05). The available phosphorus contents showed strong positive correlations with oxalic acid, malonic acid, and acetic acid (*p* < 0.01), as well as a positive correlation with citric acid (*p* < 0.05). Therefore, the LMWOA content in reclaimed soils can be improved by regulating the soil nutrient contents.

## 4. Discussion

### 4.1. Compositions and Sources of Organic Acids in Reclaimed Soil

The sources of soil organic acids are complex, resulting from the combined impacts of multiple factors, including the impacts of soil parent materials, plant species, fertilization practices, soil organic matter contents, plant root exudates, microbial synthesis, and atmospheric deposition [26,27,28,29]. Indeed, LMWOAs can be derived from different sources and accumulate in soils. These sources include plant root exudates, microbial metabolites, and organic matter decomposition, of which plant root exudates are the major source of organic acids in soils [15,30].

Plant species can secrete different LMWOA types, of which oxalic acid, citric acid, and malic acid are the dominant acids in the roots, stems, and leaves of Goji trees, mulberry trees, *orychophragmus violaceus*, and oilseed rape [30]. The root system of wild amaranth can release acetic acid, citric acid, and oxalic acid, while that of *Trifolium repens* can secrete malic acid and oxalic acid [31]. *Pinus massoniana* mainly secretes oxalic acid, tartaric acid, and malic acid, while Chinese fir mainly secretes oxalic acid and tartaric acid [32]. Citric acid, oxalic acid, and malic acid can be extracted from the rhizosphere of the chickpea [33]. Under unchanged climatic conditions and soil physicochemical properties, the dominant LMWOAs in the rhizosphere of wheat crops of the study area were lactic acid, malonic acid, and succinic acid (without considering oxalic acid and tartaric acid). The dominant LMWOAs in the *Tamarix ramosissima* root zone soil were citric acid, propionic acid, and acetic acid, while the dominant LMWOAs in the *Phragmites australis* and *Vigna radiate* root zones were tartaric acid and oxalic acid.

The release, accumulation, transport, and secretion of LMWOAs in soil play a crucial role in plants’ adaptation and environmental regulation under adverse environmental conditions [34,35]. Indeed, soil pH values can be influenced by the release of H^+^ from numerous LMWOAs (e.g., lactic acid, formic acid, malic acid, oxalic acid, and succinic acid) in plant root exudates [36,37]. These organic acids can substantially increase the H^+^ concentrations in soils, thereby decreasing the rhizosphere pH values. In this study, the reclaimed soil pH values exhibited strong significant negative correlations with the oxalic and succinic acid contents, as well as significant negative correlations with the citric and acetic acid contents. On the other hand, the fly ash used in the reclamation process increased the pH values of the reclaimed soil, while the LMWOAs effectively reduced the alkalinity of the reclaimed soils. These findings are consistent with the results revealed by Bai et al. [38] on the impact of fly ash applications on soil water retention and conditioning agents under winter wheat. The presence of organic acids in plant root exudates can lead to the acidification of the soil environment through the release of H^+^ and activate or transform insoluble soil nutrients through exchange and reduction processes, thereby decreasing organic acid contents and enhancing the available nutrient use efficiencies in soils.

The presence of LMWOAs at low contents can restrict the availability of soil phosphorus. This restriction may be due to the fact that small amounts of organic acids are conducive to soil phosphorus adsorption. The availability of soil phosphorus under the presence of LMWOAs followed the order of oxalic acid > citric acid > malic acid [39]. The accumulation of LMWOAs can enhance the release of organic matter from mineralized materials in soils, further accelerating the nitrogen mineralization process. Indeed, a previous study highlighted the key role of malic acid, citric acid, and oxalic acid in increasing soil ammonium nitrogen (NH_4_^+^−N) contents [40]. Consequently, there were strong correlations between the LMWOA and available nutrient contents in the soils.

The fly ash material had unique physical properties and relatively high available potassium and phosphorus contents. Therefore, it is important to adjust the LMWOA contents in soil to further enhance the nutrient levels in the reclaimed soil and create favorable soil environmental conditions for optimal growth of various plant species. Tartaric acid and oxalic acid were the main LMWOA types in the reclaimed soil. However, the obtained results showed significant differences in the LMWOA contents between the 0–10 and 10–40 cm soil layers. In addition, the LMWOA contents showed decreasing trends with increasing soil depth. The 0–10 cm layer had more plant roots and higher microbial activities compared with the deeper soil layers. Our results on the LMWOA variation patterns in the rhizosphere are consistent with those revealed by He et al. [41].

### 4.2. Compositions and Sources of Organic Acids in Fly Ash Material

Fly ash is fine aggregate derived from flue gas after coal combustion, consisting mostly of spherical glassy particles formed through the oxidation of coal minerals at a temperature range of 1400–1700 °C [42]. In this study, we assessed the LMWOA content in fresh fly ash samples from the Tianji Power Plant of Huaihu Coal Power Co., Ltd (Huainan, China). In addition, the LMWOA content in the fly ash−filled soil were investigated in this study. The results showed a lack of LMWOA organic in the fresh fly ash samples, demonstrating the role of high−temperature calcination in removing fly ash materials. On the other hand, seven LMWOAs were detected in the fly ash−filled soil, with only acetic acid not detected. Although the contents of the detected LMWOAs were relatively low, abundant LMWOAs were found compared with those at the soil sampling points. The LMWOA contents in the fly ash−filled soil increased after several years of filling and reclamation, which might be due to several environmental factors such as atmospheric precipitation events, trace solute transport, plant root exudate secretions, and microbial proliferation.

## 5. Conclusions

(1) Under constant climatic conditions and soil physicochemical properties, eight LMWOA types were detected in the reclaimed soil, while seven types were detected in the fly ash−filled soil. However, no LMWOAs were detected in the fresh fly ash from the power plant. According to the obtained results, the use of fresh fly ash in the reclamation process had a slight contribution to the soil organic acid content. However, the applied fly ash slightly increased the available potassium and phosphorus contents in the soil, contributing to the formation of LMWOAs.
(2) The LMWOA contents in the reclaimed soil followed the order of oxalic acid > tartaric acid > succinic acid > lactic acid > acetic acid > citric acid > propionic acid > succinic acid. Oxalic and succinic acids exhibited the highest and lowest contents of 1445.79 and 6.50 µg·g^−1^, respectively. The total LMWOA contents at the soil sampling points followed the order of farmland control soil > 1# (*Triticum aestivum*) > 4# (*Phragmites australis*) > 5# (*Vigna radiata*) > 2# (*Sorghum bicolor*) > 3# (*Tamarix ramosissima*) > fly ash−filled soil. The LMWOA contents in the reclaimed soil decreased with increasing soil depth, showing significant differences between the 0–20 and the 20–40 cm soil layers.
(3) The contents of the nutrient indicators in the reclaimed soils, including organic matter, available phosphorus, available potassium, and alkali−hydrolyzable nitrogen, were low. In contrast, the fly ash had relatively high available potassium and phosphorus contents, thereby increasing the reclaimed soil content to some extent. The results showed negative correlations between the reclaimed soil pH values and LMWOA contents (except tartaric acid). The available potassium content exhibited a strong significant positive correlation with the citric acid content, while the soil organic matter and alkali−hydrolyzable nitrogen content showed strong significant positive correlations with the lactic succinic acid content, respectively. On the other hand, the available phosphorus content showed strong significant positive correlations with the tartaric, succinic, and acetic acid content (*p* < 0.01). Therefore, the LMWOA contents in reclaimed soils can be improved by regulating the contents of soil nutrient indicators, thereby promoting the restoration of ecological functions of the rhizosphere in reclamation soil areas.
(4) The types and contents of the detected LMWOAs in the soil are influenced by several factors, including soil type, soil nutrient status, pH value, temperature, moisture content, microbial activity, and organic matter type and content. On the other hand, the LMWOAs are also affected by analytical−related factors, including detection instrument, detection method, and organic acid standard type. Therefore, the eight detected LMWOAs in the reclaimed soil in this study do not represent all soil LMWOA types. However, they can reflect the main characteristics of organic acid occurrence. Hence, future comprehensive studies on LMWOAs in reclaimed soils are required.

## Figures and Tables

**Table 1:** Comparison table of plants at sampling points.

Sampling Point	1#	2#	3#	4#	5#	6#
Botanical name	*Triticum aestivum* L.	*Sorghum bicolor* (L.) Moench	*Tamarix ramosissima*	*Phragmites australis* (Cav.) Trin. ex Steud.	*Vigna radiate* (L.) Wilczek	Farmland control soil
Growth situation	Vigorous	Vigorous	Adequate	Vigorous	Adequate	No planting

**Table 2:** Statistics of low-molecular-weight organic acid content in soil of reclamation areas (ug·g^−1^).

No.	Sample	Statistics Index	Low-Molecular-Weight Organic Acid Species							
			Tartaric Acid	Oxalic Acid	Malonic Acid	Citric Acid	Succinic Acid	Lactic Acid	Acetic Acid	Propionic Acid
1	1# *triticum aestivum*	Mean	146.43	347.81	9.52	2.44	1.46	35.07	ND	ND
		Standard deviation	12.85	86.64	5.06	1.29	0.16	24.65	/	/
		Coefficient of variation (%)	8.76	24.91	53.15	52.87	10.96	70.29	/	/
		Maximum	169.97	455.88	16.70	4.17	1.75	63.03	/	/
		Minimum	120.81	263.41	5.97	1.43	0.91	9.36	/	/
2	2# *sorghum bicolor*	Mean	33.24	94.15	3.78	2.17	0.24	ND	ND	ND
		Standard deviation	0.16	141.60	1.05	0.36	0.13	/	/	/
		Coefficient of variation (%)	0.48	110.40	27.78	16.59	54.17	/	/	/
		Maximum	51.86	312.21	9.99	2.87	0.41	/	/	/
		Minimum	24.42	29.01	1.09	1.54	0.15	/	/	/
3	3# *tamarix ramosissima*	Mean	25.96	70.95	8.18	9.71	0.98	ND	6.69	2.25
		Standard deviation	3.42	2.85	0.91	4.19	0.05	/	2.23	0.24
		Coefficient of variation (%)	13.17	4.02	11.12	43.15	5.10	/	33.33	10.67
		Maximum	39.13	95.88	19.04	32.26	1.33	/	12.99	3.69
		Minimum	13.39	50.15	4.16	2.55	0.39	/	1.38	1.12
4	4# *phragmites australis*	Mean	240.76	244.34	6.86	2.03	0.51	ND	1.75	ND
		Standard deviation	65.08	156.73	1.31	0.99	0.26	/	0.41	/
		Coefficient of variation (%)	27.03	64.14	19.10	48.77	50.98	/	23.43	/
		Maximum	441.60	467.30	12.57	3.59	0.98	/	2.20	/
		Minimum	141.45	153.85	1.22	1.06	0.20	/	1.33	/
5	5# *vigna radiata*	Mean	189.18	133.51	6.92	2.27	0.34	ND	3.27	ND
		Standard deviation	27.02	75.59	3.36	0.88	0.08	/	0.71	/
		Coefficient of variation (%)	14.28	56.61	48.55	38.77	23.53	/	21.71	/
		Maximum	238.94	199.93	12.95	4.12	0.53	/	4.16	/
		Minimum	141.75	48.76	1.34	1.14	0.18	/	2.75	/
6	Farmland control soil	Mean	60.81	522.35	23.78	2.24	2.39	ND	36.65	ND
		Standard deviation	4.35	86.61	3.24	1.34	0.09	/	65.49	/
		Coefficient of variation (%)	7.15	16.58	13.62	59.82	3.76	/	128.69	/
		Maximum	90.96	695.34	33.77	4.17	2.73	/	132.46	/
		Minimum	38.77	390.18	14.08	1.43	2.17	/	1.48	/
7	Fly ash-filled soil	Mean	19.43	32.68	6.96	4.56	0.58	13.45	ND	7.43
8	Power plant fly ash fresh	Mean	ND	ND	ND	ND	ND	ND	ND	ND

**Table 3:** Soil physicochemical indexes.

No.	Land Types	Solum (cm)	pH	Organic Matter (g·kg^−1^)	Available Potassium (mg·kg^−1^)	Available Phosphorus (mg·kg^−1^)	Alkali−Hydrolyzable Nitrogen (mg·kg^−1^)
1	Reclamation area soil	0–10	7.27 ± 0.22	24.73 ± 0.11	121.77 ± 0.21	9.40 ± 0.15	60.36 ± 1.70
		10–20	7.47 ± 0.17	19.56 ± 0.03	120.27 ± 0.04	7.29 ± 0.34	56.94 ± 0.32
		20–30	7.80 ± 0.02	17.13 ± 0.38	118.13 ± 0.35	5.89 ± 0.09	48.87 ± 0.77
		30–40	7.82 ± 0.11	13.01 ± 0.21	76.22 ± 0.01	5.58 ± 0.01	42.33 ± 0.15
2	Farmland control soil	0–10	6.57 ± 0.14	17.88 ± 0.17	146.38 ± 0.11	20.90 ± 1.98	87.22 ± 3.14
		10–20	7.19 ± 0.03	12.61 ± 0.20	144.93 ± 0.06	19.23 ± 0.05	80.23 ± 3.28
		20–30	7.12 ± 0.13	10.43 ± 0.09	105.95 ± 0.33	17.70 ± 1.14	90.16 ± 3.51
		30–40	7.41 ± 0.11	9.28 ± 0.07	98.22 ± 0.12	11.88 ± 0.09	76.86 ± 0.01
3	Fly ash−filled soil		10.58 ± 0.06	18.10 ± 0.67	145.02 ± 2.34	58.95 ± 2.31	30.10 ± 4.90

**Table 4:** Correlation coefficient of soil organic acid and soil nutrient, pH values.

No.	Nutrient Index	Tartaric Acid	Oxalic Acid	Malonic Acid	Lactic Acid	Citric Acid	Ssuccinic Acid	Propionic Acid	Acetic Acid
1	pH	0.124	−0.802 **	−0.889 **	−0.218	−0.423 *	−0.37	−0.126	−0.424 *
2	Available potassium	−0.508 *	−0.638 **	−0.085	0.149	0.708 **	0.217	0.258	−0.316
3	Organic matter	−0.179	0.393	0.488 *	0.648 **	0.510 *	0.295	0.172	0.316
4	Alkali−hydrolyzable nitrogen	−0.559 **	0.411 *	0.607 **	−0.111	0.397	0.176	0.001	0.371
5	Available phosphorus	−0.115	0.679 **	0.710 **	−0.049	0.453 *	−0.051	−0.193	0.644 **

Note: “*” indicates significant correlation (*p* < 0.05), “**” indicates extremely significant correlation (*p* < 0.01).

## Data Availability

The datasets used and/or analyzed during the current study are available from the corresponding author upon reasonable request.
